# Predictors of low cardiac output after isolated pericardiectomy: an observational study

**DOI:** 10.1186/s13741-022-00267-y

**Published:** 2022-08-17

**Authors:** Jin Wang, Xiaohong Zhang, Xingrong Liu, Lijian Pei, Yuelun Zhang, Chunhua Yu, Yuguang Huang

**Affiliations:** 1 Department of Anesthesiology, Peking Union Medical College Hospital, Chinese Academy of Medical Sciences, Beijing, China; 2 Department of Anesthesiology, Shengli Clinical Medical College of Fujian Medical University, Fujian Provincial Hospital, Fuzhou, China; 3 Department of Cardiac Surgery, Peking Union Medical College Hospital, Chinese Academy of Medical Sciences, Beijing, China; 4 Department of Biostatistics and Epidemiology, Peking Union Medical College Hospital, Chinese Academy of Medical Sciences, Beijing, China

**Keywords:** Low cardiac output, Pericardiectomy, Constrictive pericarditis

## Abstract

**Background:**

Low cardiac output is the main cause of perioperative death after pericardiectomy for constrictive pericarditis. We investigated the associated risk factors and consequences.

**Methods:**

We selected constrictive pericarditis patients undergoing isolated pericardiectomy from January 2013 to January 2021. Postoperative low cardiac output was defined as requiring mechanical circulatory support or more than one inotrope to maintain a cardiac index > 2.2 L •min^−1^ •m^−2^ without hypoperfusion, despite adequate filling status. Uni- and multivariable analysis were used to identify factors associated with low cardiac output. Cox regression was used to identify factors associated with length of hospital stay.

**Results:**

Among 212 patients with complete data, 55 (25.9%) developed low cardiac output within postoperative day 1 (quartiles 1 and 2), which caused seven of the nine perioperative deaths. The rates of atrial arrhythmia, renal dysfunction, hypoalbuminemia, modest-to-severe hyponatremia, and hyperbilirubinemia caused by constrictive pericarditis were 9.4%, 12.3%, 49.1%, 10.4%, and 81.6%. The mean preoperative central venous pressure and cardiac index were 18 ± 5 cmH_2_O and 1.87 ± 0.45 L•min^−1^•m^−2^. Univariable analysis showed that low cardiac output patients had higher rates of atrial arrhythmia (OR 3.32 [1.35, 8.17], *P = 0.007*), renal dysfunction (OR 4.24 [1.94, 9.25], *P < 0.001*), hypoalbuminemia (OR 1.99 [1.06, 3.73], *P = 0.031*) and hyponatremia (OR 6.36 [2.50, 16.20], *P < 0.001*), greater E peak velocity variation (difference 2.8 [0.7, 5.0], *P* = 0.011), higher central venous pressure (difference 3 [2,5] cmH_2_O, *P < 0.001*) and lower cardiac index (difference − 0.27 [− 0.41, − 0.14] L•min^−1^•m^−2^, *P < 0.001*) than patients without low cardiac output. Multivariable regression showed that atrial arrhythmia (OR 4.04 [1.36, 12.02], *P* = 0.012), renal dysfunction (OR 2.64 [1.07, 6.50], *P* = 0.035), hyponatremia (OR 3.49 [1.19, 10.24], *P* = 0.023), high central venous pressure (OR 1.17 [1.08, 1.27], *P* < 0.001), and low cardiac index (OR 0.36 [0.14, 0.92], *P* = 0.032) were associated with low cardiac output (AUC 0.79 [0.72–0.86], *P* < 0.001). Cox regression analysis showed that hyperbilirubinemia (HR 0.66 [0.46, 0.94], *P* = 0.022), renal dysfunction (HR 0.51 [0.33, 0.77], *P* = 0.002), and low cardiac output (HR 0.42 [0.29, 0.59], *P* < 0.001) were associated with length of hospital stay.

**Conclusions:**

Early recognition and management of hyponatremia, renal dysfunction, fluid retention, and hyperbilirubinemia may benefit constrictive pericarditis patients after pericardiectomy.

## Background

Pericardiectomy is the most common and only definitive treatment for constrictive pericarditis (Gopaldas et al. 2013; Tokuda et al. 2013; Schumann et al. 2018; Adler et al. 2015). Low cardiac output is one of the main complications and causes of perioperative death after pericardiectomy for this group of patients (Gillaspie et al. 2016; Zhu et al. 2015; Busch et al. 2015; Szabo et al. 2013; Chowdhury et al. 2006; Ling et al. 1999). With various concomitant surgeries, the incidence of low cardiac output ranges from 3 to 59.6%, and it accounts for 33–100% of perioperative deaths (Gillaspie et al. 2016; Zhu et al. 2015; Ling et al. 1999; Bertog et al. 2004).

Several studies have been conducted to investigate factors influencing the short- and long-term survival rates of patients who undergo pericardiectomy; however, these studies either had small sample sizes or included heterogeneous patients with various etiologies or those who underwent combined valve or coronary artery procedures (Zhu et al. 2015; Busch et al. 2015; Szabo et al. 2013; Murashita et al. 2017; Kang et al. 2014). The current literature does not include any study focusing on the clinical features and preoperative predictors of low cardiac output in constrictive pericarditis patients receiving isolated pericardiectomy. An understanding of the pathogenesis and clinical features of this group of patients may facilitate early prediction, detection and treatment, thus improving patient outcomes.

This single-center observational study investigated the clinical features and preoperative predictors of patients who developed low cardiac output after pericardiectomy for constrictive pericarditis. Perioperative clinical features were compared between patients with and without postoperative low cardiac output, and the predictors were selected based on the comparison results.

## Methods

### Study design

This was a single center, observational study with the approval of the hospital’s institutional review board (No. S-K948). No informed consent was required because the data were anonymized.

### Settings and participants

The study was conducted in a tertiary hospital in Beijing, China. Patients’ medical data were prospectively entered into an electronic database, which was managed by a dedicated data coordination team. The following inclusion criterion was used: patients who underwent isolated pericardiectomy for constrictive pericarditis from January 2013 to January 2021. The following exclusion criteria were used: patients who underwent concomitant cardiac procedures, such as coronary artery bypass grafting, valve surgeries, and repeat pericardiectomy.

### Variables and definitions

The essential variables that were collected included preoperative comorbidities, clinical manifestations, echocardiography, hemodynamic parameters, postoperative complications, and outcomes.

#### Preoperative data

Preoperative comorbidities were collected from the past medical histories and preoperative examination results of the patients. The major comorbidities included hypertension, diabetes mellitus, coronary artery disease, myocardial infarction, cerebral infarction, and chronic kidney disease. The distinctive clinical manifestations that were collected included signs of fluid overload and major organ dysfunction, including cardiac, hepatic, and renal dysfunction. Peripheral edema was determined by physical examination. Pleural effusion, ascites, and pericardial calcification were determined from the ultrasound and computed tomography imaging results. Atrial arrhythmia, including atrial flutter and fibrillation, was determined from the current medical history and electrocardiogram. Biochemical disturbances and hepatic and renal dysfunctions (serum creatinine > 1.2 mg/dL in males and > 1.1 mg/dL in females) were determined from the blood test results. Biochemical disturbances included moderate to severe hypokalemia (serum potassium < 3 mmol/L), moderate to severe hyponatremia (serum sodium < 130 mmol/L) and hypoalbuminemia (albumin < 3.5 g/dL). Hepatic dysfunction included hyperbilirubinemia, hepatomegaly, and coagulopathy. Hepatomegaly was determined from ultrasound results. The test results of patients with prior uses of anticoagulation medication were excluded from the coagulopathy evaluation. The transthoracic echocardiography parameters collected included left ventricular ejection fraction (LVEF), tricuspid regurgitation flow rate, tricuspid annular plane systolic excursion (TAPSE), the E/A ratio, inferior vena cava diameter, and E peak velocity variation. The hemodynamic parameters collected included central venous pressure and cardiac index. Central venous pressure was measured via a central venous catheter, and cardiac index was measured by using the transpulmonary thermodilution method with a pulse index continuous cardiac output device (PV 8215, PULSION Medical system SE. Corp., Germany).

#### Outcomes and definitions

All pericardiectomies were performed by one team consisting of five surgeons. All patients were placed in the supine position and draped in the standard fashion for cardiac surgery. Pericardiectomy was performed through conventional median sternotomy, on beating heart without cardiopulmonary bypass. An anterior midline pericardial incision was first made by using sharp dissection to find a proper dissection plane between the stiffened parietal pericardium and the epicardial adipose tissue. Then, the dissection was extended bilaterally along this plane, both to the right and left chamber walls. On both sides, pericardiectomy initially ended 0.5 cm anterior to the phrenic nerve. However, on the left side, pericardiectomy was resumed from 0.5 cm posterior to the phrenic nerve, extending beyond the left atrioventricular groove. Pericardium covering the superior and inferior cavoatrial junctions was removed carefully as well. After complete pericardiectomy was obtained, hemostasis was achieved, and thoracic drains were inserted before the chest was closed.

Postoperatively, low cardiac output syndrome was defined as follows: (1) despite adequate filling status, patients required more than one inotrope to maintain a persistent systolic blood pressure > 90 mmHg, a cardiac index > 2.2 L•min^−1^•m^−2^ and no signs of tissue hypoperfusion; or (2) patients required the use of mechanical circulatory support devices, such as an intra-aortic balloon pump (IABP) or extracorporeal membrane oxygenation (ECMO) during or after the surgery (Lomivorotov et al. 2017; Maganti et al. 2005; Ponikowski et al. 2016; Ibanez et al. 2018; Subspecialty Group of A, Intensive cardiac care of Chinese Society of C, Editorial Board of Chinese Journal of C 2019; van Diepen et al. 2017).

The postoperative complications that were collected included acute kidney injury, tachyarrhythmia, delirium and new-onset chronic renal dysfunction. Acute kidney injury and chronic renal dysfunction were determined according to the Kidney Disease Improving Global Outcomes (KDIGO) criteria (Kellum and Lameire 2013; Palevsky et al. 2013; Inker et al. 2014). Patients with chronic renal dysfunction before pericardiectomy were excluded from calculation of new-onset chronic renal dysfunction. Tachyarrhythmia included new onset atrial fibrillation, atrial fibrillation with a rapid ventricular rate and supraventricular and ventricular tachycardia in the absence of electrolyte abnormalities. Delirium was evaluated by using the Confusion Assessment Method-Intensive Care Unit score every twelve hours and at times when delirium was suspected (Ely et al. 2001; Gusmao-Flores et al. 2012).

Uses of mechanical circulatory support devices, including IABP, ECMO, and hemofiltration, were also recorded. Other outcomes included ventilator hours, length of intensive care unit (ICU) and hospital stays and mortality. Mortality was defined as any postoperative death that occurred during the same hospital admission or within 30 days after discharge. The cause of death was discussed, and a clinical judgement was made by clinicians responsible for the patient’s perioperative care. Follow-up was performed until 6 months after the operation. Patient status was determined from either a clinical visit or a telephone call.

### Statistical analysis

Statistical analysis was performed with SPSS 24.0.0.0 software (IBM Corp). Normality was tested with a Q-Q plot. Continuous variables with a normal distribution are expressed as the mean ± standard deviation; additionally, continuous variables with a non-normal distribution are expressed as medians (quartile), and categorical variables are expressed as case numbers and percentages. An independent *t* test was performed to analyze the continuous variables with a normal distribution. The Mann-Whitney *U* test was used for the analysis of the continuous variables with a non-normal distribution. The chi-square test was used to evaluate categorical data when the expected cell counts were > 5; otherwise, Fisher’s exact test was used.

Preoperative variables that were considered to be related to low cardiac output, including atrial fibrillation, hyponatremia, hypoalbuminemia, renal dysfunction, E peak velocity variation, central venous pressure, and cardiac index were selected for multivariable logistic regression analysis. Calibration was assessed with the Hosmer-Lemeshow goodness-of-fit statistic. Model discrimination was evaluated using the area under the receiver operating characteristic curve. The log-rank test and Cox regression were used to identify independent factors associated with length of hospital stay. All of the tests were two-tailed, and a *P* value < 0.05 was considered to be statistically significant.

## Results

### Participants and descriptive data

From January 2013 to January 2021, a total of 254 patients underwent pericardiectomy for constrictive pericarditis in the designated hospital. Nineteen combined operations were excluded from the study, including ten coronary artery bypass grafting surgeries, six valve repairs/replacements, two tumor resections and one ventricular aneurysm repair. Twenty-three patients missing part of the data or lacking defined outcome data were included in the total analysis (Tables 1, 2, and 3, “Total”) but not in the group analysis (Tables 1, 2, and 3, “LCO and non-LCO”, Tables 4 and 5). A total of 212 constrictive pericarditis patients underwent isolated pericardiectomy were included in univariate and multivariate regression analysis.

**Table 1:** Baseline data of constrictive pericarditis patients with and without low cardiac output after pericardiectomy

	Total (***n*** = 235)	LCO (***n*** = 55)	Non-LCO (***n*** = 157)	***P*** value	OR/difference (95% CI)
***Demographics***					
Male (*n*, %)	168, 71.5%	39, 70.9%	117, 74.5%	*0.601*	0.83 (0.42, 1.65)
Age (years/old)	46 ± 16	48 ± 17	45 ± 16	*0.120*	4 (-1,9)
BMI (kg•m^−2^)	22.31±3.99	22.11±3.72	22.35±4.11	*0.704*	-0.24 (-1.49, 1.00)
BSA (m^2^)	1.78 ± 0.21	1.75 ± 0.19	1.79±0.22	*0.238*	-0.04 (-0.10,0.03)
Smoking (*n*, %)	67, 31.6%	18, 32.7%	49, 31.2%	*0.767*	1.11 (0.57, 2.13))
Alcohol use (*n*, %)	38, 17.9%	11, 20%	27, 17.2%	*0.641*	1.20 (0.55, 2.63)
***Comorbidities (n, %)***					
Hypertension	42, 17.9%	9, 16.4%	30, 19.1%	*0.651*	0.83 (0.37, 1.88)
Diabetes mellitus	22, 9.4%	6, 10.9%	14, 8.9%	*0.664*	1.25(0.46, 3.43)
CAD	30, 12.8%	8, 14.5%	20, 12.7%	*0.733*	1.17 (0.48, 2.82)
MI	4, 1.7%	0, 0.0%	4, 2.5%	*0.999*	0.55 (0.06, 4.81)
Cerebral infarction	9, 3.8%	2, 3.6%	7, 4.5%	*0.999*	0.81 (0.16, 4.02)
CKD	7, 3.0%	2, 3.6%	4, 2.5%	*0.651*	1.44 (0.26, 8.11)

**Table 2:** Preoperative features of constrictive pericarditis patients with and without low cardiac output after pericardiectomy

Clinical features ***n***, %	Total (***n*** = 235)	LCO (***n*** = 55)	Non-LCO (***n*** = 157)	***P*** value	OR (95% CI)
Atrial arrhythmia	27, 11.5%	11, 20.0%	11, 7.0%	0.007^*^	3.32 (1.35, 8.17)
Pleural effusion	178, 84.0%	48, 87.3%	130, 82.8%	*0.437*	1.42 (0.58, 3.49)
Ascites	155, 73.1%	44, 80%	111, 70.7%	*0.181*	1.66 (0.79, 3.49)
Peripheral edema	183, 86.3%	47, 85.5%	136, 86.6%	*0.828*	0.91 (0.38, 2.19)
Anemia	50, 23.6%	14, 25.5%	36, 22.9%	*0.704*	1.15 (0.56, 2.34)
Renal dysfunction	38,16.1%	17, 30.9%	15, 9.6%	< 0.001^*^	4.24 (1.94, 9.25)
Hypoalbuminemia	121, 47.8%	34, 61.8%	70, 44.6%	0.031^*^	1.99 (1.06, 3.73)
Hyponatremia	23, 10.6%	14. 25.5%	8, 5.1%	< 0.001^*^	6.36 (2.50, 16.20)
Hypokalemia	26, 10.3%	9, 16.4%	14, 8.9%	*0.126*	2.00 (0.81, 4.92)
Hepatomegaly	115, 54.2%	30, 54.5%	85, 54.1%	*0.959*	1.02 (0.55, 1.88)
Hyperbilirubinemia	173, 81.2%	46, 83.6%	127, 80.9%	*0.651*	1.21 (0.53, 2.74)
Coagulopathy	157, 74.1%	45, 81.8%	112, 71.3%	*0.127*	1.81 (0.84, 3.90)
Calcification	46, 21.7%	13, 23.6%	33, 21.0%	*0.685*	1.16 (0.56, 2.42)
LVEF (%)	64 ± 7	64 ± 9	64 ± 7	*0.981*	0 (− 3,3)
TR (m/s)	2.16 ± 0.32	2.19 ± 0.39	2.15 ± 0.30	*0.462*	0.04 (− 0.07, 0.14)
TAPSE (mm)	13 ± 4	12 ± 4	13 ± 3	*0.672*	− 1 (− 6,4)
IVC diameter(mm)	23 ± 3	23 ± 3	23 ± 3	*0.914*	0 (− 1,1)
E peak velocity variation (%)	29.6 ± 7.2	31.5 ± 7.5	28.7 ± 6.8	0.011^*^	2.8 (0.7,5.0)
E/A	1.5 ± 0.5	1.3 ± 0.5	1.5 ± 0.5	*0.076*	− 0.2 (− 0.4, 0.0)
CVP (cmH_2_O)	18 ± 5	20 ± 5	17 ± 4	< 0.001^*^	3 (2,5)
CI (L•min^−1^•m^−2^)	1.87 ± 0.45	1.67 ± 0.49	1.94 ± 0.42	< 0.001^*^	− 0.27 (− 0.41, − 0.14)

*Statistically significant difference

**Table 3:** Postoperative complications and outcomes between patients with and without low cardiac output

Postoperative outcomes (*n*, %)	Total (***n*** = 235)	LCO (***n*** = 55)	Non-LCO (***n*** = 157)	***P*** value	OR (95% CI)
Tachyarrhythmia	46, 21.7%	28, 50.9%	18, 11.4%	< 0.001^*^	8.01(3.89, 16.48)
AKI	92, 43.4%	42, 76.4%	50, 31.8%	< 0.001^*^	6.91(3.41, 14.02)
New-onset CKD	6, 2.6%	6, 10.9%	0, 0.0%	< 0.001^*^	22.12 (2.66,184.15)
Delirium	26, 12.3%	20, 36.4%	6, 3.8%	< 0.001^*^	14.38(5.38, 38.46)
Hemofiltration	35, 14.9%	25, 45.5%	5, 3.2%	< 0.001^*^	25.33 (8.98, 71.46)
IABP	8, 3.4%	7, 12.7%	0, 0.0%	< 0.001^*^	25.80 (3.15, 211.37)
ECMO	7, 3.0%	6, 10.9%	0, 0.0%	< 0.001^*^	22.12 (2.66, 184.15)
Ventilator (hrs)	30 (18, 84)	142(75, 272)	24 (15, 45)	< 0.001^*^	104 (69, 135)
ICU stay (days)	3 (2, 6)	11 (5,17)	2 (2, 4)	< 0.001^*^	7 (5, 10)
LOS (days)	24 (17, 34)	34 (25, 59)	22 (16, 28)	< 0.001^*^	13 (8, 18)
Mortality	11, 4.7%	9, 16.4%	0, 0.0%	< 0.001^*^	33.62(4.19, 269.43)

*Statistically significant difference

**Table 4:** Multivariable logistic regressions on factors associated with low cardiac output

	***B***	S.E.	OR	95% CI OR	***P*** value
Atrial arrhythmia	1.40	0.56	4.04	1.36, 12.01	0.012^*^
Renal dysfunction	0.97	0.46	2.64	1.07, 6.50	0.035^*^
Hyponatremia	1.25	0.55	3.49	1.19, 10.24	0.023^*^
Central venous pressure	0.15	0.04	1.17	1.08, 1.27	< 0.001^*^
Cardiac index	− 1.03	0.48	0.36	0.14, 0.92	0.032^*^

*Statistically significant difference

**Table 5:** Cox regression of factors associated with length of hospital stay

	***B***	S.E.	HR	95% CI HR	***P*** value
Renal dysfunction	− 0.68	0.21	0.51	0.33, 0.77	0.002^*^
Hyperbilirubinemia	− 0.42	0.18	0.66	0.46, 0.94	0.022^*^
Post-op LCO	− 0.88	0.18	0.42	0.29, 0.59	< 0.001^*^

*Statistically significant difference

## Main results

### Cohort characteristics

Among the 212 patients with complete data, 55 (25.9%) developed low cardiac output within postoperatives day 1 (quartiles 1 and 2). The baseline characteristics and comorbidities were similar between the patients with and without low cardiac output (Table 1).

### Preoperative factors associated with low cardiac output

The following analysis was based on the 212 complete data points unless otherwise stated. Preoperatively, 22 (10.4%) patients had atrial arrhythmia, and 21 (9.4%) cases were considered to be due to constrictive pericarditis-induced high atrial pressure. Thirty-two (15.1%) patients had renal dysfunction, and 26 (12.3%) cases were considered to be due to constrictive pericarditis-induced prerenal insufficiency. The overall rates of hypoalbuminemia, moderate to severe hyponatremia, hyperbilirubinemia, and pericardial calcification were 49.1%, 10.4%, 81.6%, and 21.7%, respectively. The mean central venous pressure was 18 ± 5 cmH_2_O, and the cardiac index was 1.87 ± 0.45 L•min^−1^•m^−2^.

For the between-group comparison, postoperative low cardiac output patients had higher rates of atrial arrhythmia (OR 3.32, 95%CI 1.35–8.17, *P =* 0.007), renal dysfunction (OR 4.24, 95%CI 1.94–9.25, *P* < 0.001), moderate to severe hyponatremia (OR 6.36, 95%CI 2.50–16.20, *P* < 0.001), and hypoalbuminemia (OR 1.99, 95%CI 1.06–3.73, *P = 0.031*) than patients without postoperative low cardiac output (Table 2). For echocardiography, the E peak velocity variation was greater for patients with postoperative low cardiac output than for those without (difference 2.8%, 95%CI 0.7–5.0%, *P* = 0.011). Other parameters, including LVEF, tricuspid regurgitation flow rate, TAPSE, diameter of the inferior vena cava, and E/A ratio, were similar between the two groups (all *P* >0.05). For hemodynamic parameters, postoperative low cardiac output patients had higher preoperative central venous pressure (difference 3 cmH_2_O, 95%CI 2–5 cmH_2_O, *P* < 0.001) and lower preoperative cardiac index (difference − 0.27 L•min^−1^•m^−2^, 95%CI − 0.41 to − 0.14 L•min^−1^•m^−2^, *P* < 0.001) than patients without postoperative low cardiac output (Table 2).

**Fig. 1:**
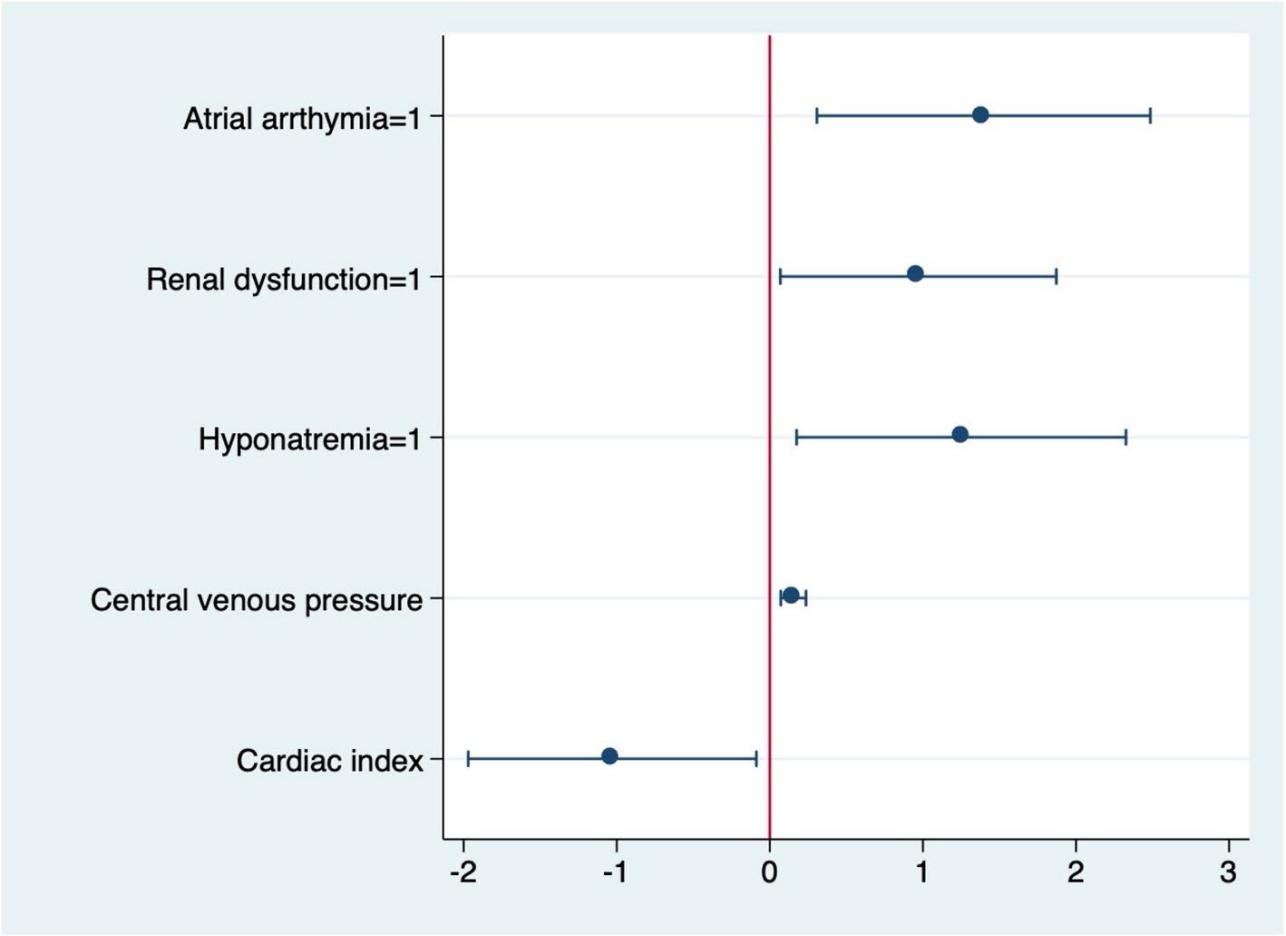
Factors associated with low cardiac output after pericardiectomy

**Fig. 2:**
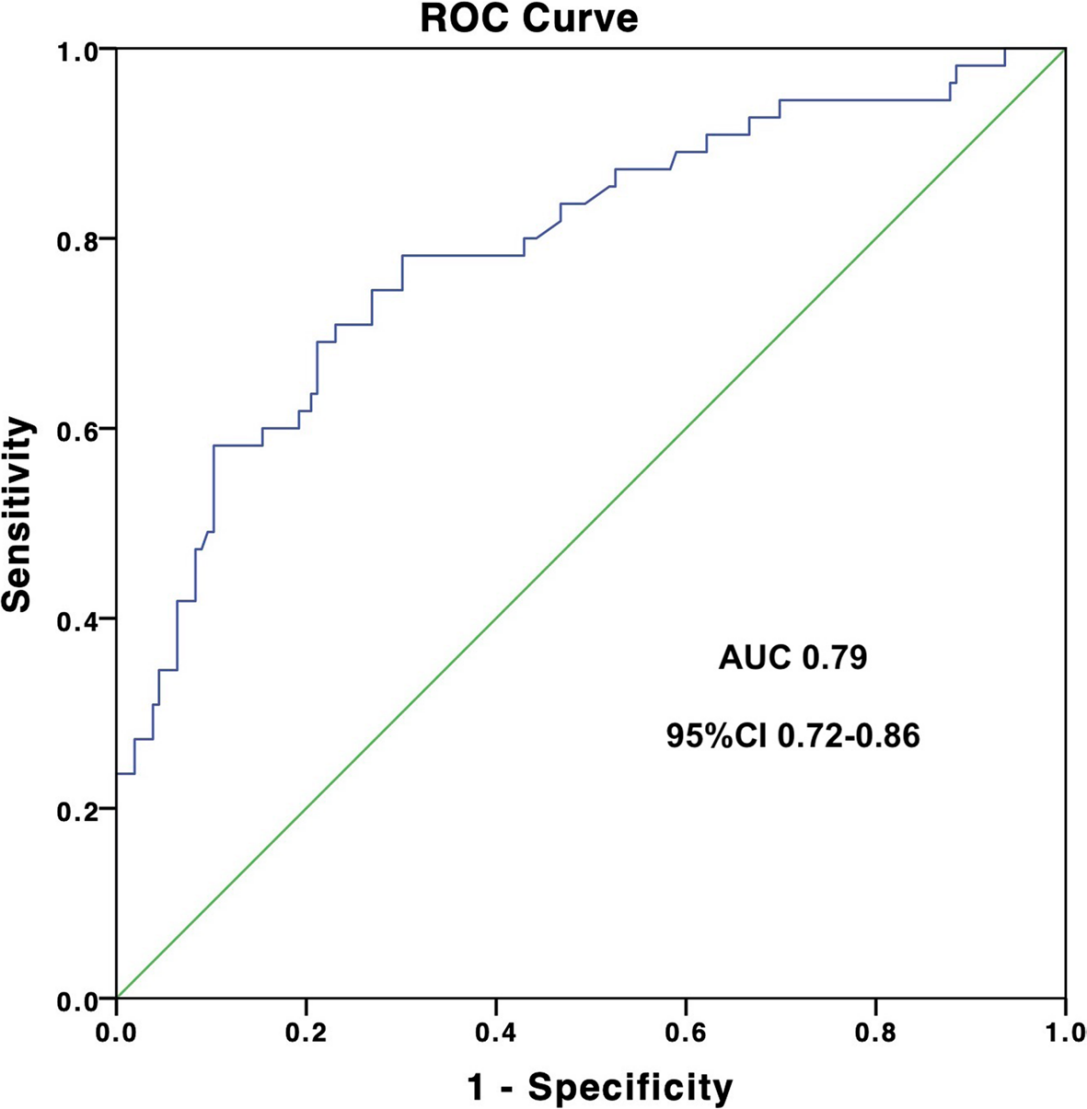
Receiver-operating-curve of the model predicting low cardiac output after pericardiectomy

The multivariable logistic regression test results showed that the preoperative factors associated with postoperative low cardiac output included atrial arrhythmia (*B* 1.40, OR 4.04, 95%CI 1.36–12.01, *P* = 0.012), renal dysfunction (*B* 0.97, OR 2.64, 95%CI 1.07–6.50, *P* = 0.035), moderate to severe hyponatremia (*B* 1.25, OR 3.49, 95%CI 1.19–10.24, *P* = 0.023), high central venous pressure (*B* 0.15, OR 1.17, 95%CI 1.08–1.27, *P* < 0.001), and low cardiac index (*B* − 1.03, OR 0.36, 95%CI 0.14–0.92, *P* = 0.032), and the results showed good model fitness (Hosmer-Lemeshow test *P* = 0.502) and an area under the curve value of 0.79 (95% CI 0.72–0.86, *P*< 0.001) (Table 4 and Figs. 1 and 2).

### The effects of low cardiac output on outcomes

Postoperatively, low cardiac output patients had higher rates of complications, including tachyarrhythmia (OR 8.01, 95%CI 3.89–16.48, *P* < 0.001), acute kidney injury (OR 6.91, 95%CI 3.41–14.02, *P* < 0.001), new-onset chronic renal dysfunction (OR 22.12, 95%CI 2.66–184.15, *P* < 0.001), and delirium (OR 14.38, 95%CI 5.38–38.46, *P* < 0.001); additionally, these patients used more circulatory support devices, including hemofiltration (OR 25.33, 95%CI 8.98–71.46, *P* < 0.001), IABP (OR 25.80, 95%CI 3.15–211.37, *P* < 0.001), and ECMO (OR 22.12, 95%CI 2.66–184.15, *P* < 0.001), and they had poorer outcomes, including longer ventilator hours (difference 104 h, 95%CI 69–135 h, *P* < 0.001), lengths of ICU (difference 7 days, 95%CI 5–10 days, *P* < 0.001), and hospital (difference 13 days, 95%CI 8–18 days, *P* < 0.001) stays, and higher mortality (OR 33.62, 95%CI 4.19–269.43, *P* < 0.001) (Table 3).

Among the 235 patients, eleven (4.7%) died perioperatively. Nine of them died in the hospital due to intractable low cardiac output. Two patients abandoned treatment and were discharged from the ICU for non-medical reasons. One of them died of multiple organ dysfunction within hours, and the other died of an unknown cause within days.

**Fig. 3:**
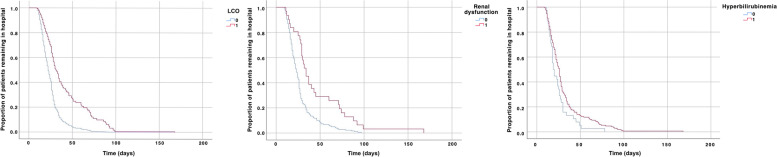
Independent factors associated with length of hospital stay

A total of 212 patients were analysed to identify independent factors associated with length of hospital stay. Preoperative renal dysfunction (*B* − 0.68, HR 0.51, 95%CI 0.33–0.77, *P* = 0.002), hyperbilirubinemia (*B* − 0.42, HR 0.66, 95%CI 0.46–0.94, *P* = 0.022), and postoperative low cardiac output (*B* −0.88, HR 0.42, 95%CI 0.29–0.59, *P* < 0.001) were associated with the length of hospital stay (Table 5 and Fig. 3).

## Discussion

### Key results

This study investigated the clinical features of constrictive pericarditis patients who underwent pericardiectomy and compared the differences between patients with and without postoperative low cardiac output. The incidence of low cardiac output was 25.9%, and mortality was 4.7% in the studied population. Nine (81.8%) of the 11 deaths were caused by low cardiac output. Multivariable regression results showed that preoperative renal dysfunction, atrial arrhythmia, moderate to severe hyponatremia, high central venous pressure, and low cardiac index were associated with a higher risk of low cardiac output after pericardiectomy for constrictive pericarditis. In addition to low cardiac output, preoperative renal dysfunction and hyperbilirubinemia were also associated with a longer length of hospital stay.

### Interpretation

Few publications have reported factors associated with low cardiac output after isolated pericardiectomy for constrictive pericarditis. There were three relatively large studies on patients undergoing concomitant or isolated pericardiectomy in different historical periods or due to different etiologies, which depicts an important general picture for this group of patients. Murashita et al. reviewed patients undergoing various types of pericardiectomy, including sternotomy, thoracotomy, and concomitant valve or coronary artery surgeries, and found that the long-term survival rate decreased from 59.4% in the historical era (median follow-up 9.7 years) to 46% in the contemporary era (median follow-up 2.4 years) (Murashita et al. 2017). However, no data on heart failure after pericardiectomy have been reported. Gillaspie et al. studied the outcomes of patients undergoing isolated pericardiectomy for either inflammatory effusive pericarditis (30.8%) or constrictive pericarditis (69.2%) (Gillaspie et al. 2016). They found that univariate predictors that were associated with an increased risk of early death included lower LVEF and preoperative renal insufficiency; however, no multivariate regression analysis were performed due to the insufficient number of events. Gopaldas et al. studied a large group of patients undergoing pericardiectomy due to constrictive pericarditis (28%), pericardial calcification (15%), secondary malignancies (3%), adhesive pericarditis (2%) and other causes (40%). They found that the average in-hospital complication and mortality rates were 48.2% and 7.5%, respectively (Gopaldas et al. 2013). The overall complication rates and mortality were associated with age, sex, race, etiology, and comorbidities. No specific high-risk comorbidity was identified because the study analyzed the Charlson-Deyo comorbidity index instead of each specific comorbidity. In addition, Sabzi et al. summarized the clinical data of pericardial effusion and constrictive pericarditis patients undergoing either pericardiotomy or pericardiectomy. The results showed that malignancy, radiotherapy, low ejection fraction, calcified pericardium, and connective tissue disease were associated with low cardiac output after pericardiotomy (Sabzi and Faraji 2015).

Our study investigated patients undergoing isolated pericardiectomy for constrictive pericarditis. The results showed that preoperative fluid retention, hyponatremia, and poor renal and cardiac function were associated with a high risk of low cardiac output. Fluid retention is one of the core features of constrictive pericarditis. According to a previous study, the total body fluid of constrictive pericarditis patients increased by 36% and primarily occurred in the extracellular space (81%) (Anand et al. 1991). The underlying mechanism was considered to be constrictive diastolic filling dysfunction, which not only increases venous pressure but also induces systematic hormone disturbances, including impaired secretion of atrial natriuretic factor and stimulation of the renin-angiotensin-aldosterone system (Svanegaard et al. 1990; Anand et al. 1989). A high central venous pressure before pericardiectomy implies more severe fluid retention in both the intravascular and extravascular spaces. After pericardiectomy, a dramatic fluid return from the extravascular space into the intravascular space occurs, which is driven not only by the relief of mechanical restriction but also by the restoration of the hormonal natriuretic and diuretic effects. If cardiac function is too poor to adapt to the volume shift, then heart failure can occur.

Renal dysfunction is also an important outcome indicator, since 81% of the patients with preoperative renal dysfunction did not have a previous renal disease history, and their kidney injury was considered to be the result of constrictive pericarditis-induced prerenal insufficiency. According to a previous study, renal plasma flow decreased by 49% in this group of patients (Anand et al. 1991). The underlying mechanism was considered to be reduced renal perfusion due to decreased arterial pressure and increased venous pressure. Efforts should be made to improve these factors. Early use of inotropes may be considered, and the timing and dosages of diuretics should be chosen carefully to reduce preload as much as possible, meanwhile, not to induce intravascular depletion or severe electrolyte disturbance.

Additionally, in patients with a history of long-term constrictive pericarditis, myocardial atrophy and ventricular re-modelling may gradually develop, and 20–40% of them also had atrial arrhythmia, which predisposes them to intractable low cardiac output (Adler et al. 2015; Bertog et al. 2004; Choudhry et al. 2015; Welch 2018; Schwefer et al. 2009). For these patients, preoperative cardiac magnetic resonance imaging may be considered to evaluate myocardial involvement.

This study had some limitations. First, this is a single-center observational study and suffers from all of the shortcomings of this type of study. Second, the study results applied only to patients undergoing isolated pericardiectomy. Therefore, patients undergoing combined valve or coronary artery surgeries may have completely different clinical pictures, and their management requires further investigation.

## Conclusion

This study investigated the clinical features of constrictive pericarditis patients who developed low cardiac output after pericardiectomy. The results showed that preoperative atrial arrhythmia, renal dysfunction, modest to severe hyponatremia, high central venous pressure, and low cardiac index were associated with an increased risk of low cardiac output after isolated pericardiectomy for constrictive pericarditis. The model may help clinicians in the early prediction, detection and management of cardiac dysfunction, as well as improve prognosis.

## Data Availability

The datasets used and/or analyzed during the current study are available from the corresponding author on reasonable request.

## Abbreviations

LCO: Low cardiac output
BMI: Body mass index
BSA: Body surface area
CAD: Coronary artery disease
MI: Myocardial infarction
CKD: Chronic kidney disease
CVP: Central venous pressure
CI: Cardiac index
AKI: Acute kidney injury
IABP: Intra-aortic balloon pump
ECMO: Extracorporal membrane oxygenation
ICU: Intensive care unit
LOS: Length of hospital stay
AUC: Area under the curve
LVEF: Left ventricle ejection fraction
E/A: E versus A ratio
TR: Tricuspid regurgitation flow rate
IVC: Inferior vena cava
TAPSE: Tricuspid annular plane systolic excursion
